# Quality-of-life: a many-splendored thing? Belgian population norms and 34 potential determinants explored by beta regression

**DOI:** 10.1007/s11136-017-1556-y

**Published:** 2017-03-27

**Authors:** Joke Bilcke, Niel Hens, Philippe Beutels

**Affiliations:** 1 Centre for Health Economics Research and Modeling Infectious Diseases (CHERMID), Vaccine and Infectious Disease Institute (VAXINFECTIO), University of Antwerp - CDE R2.07, 2610 Wilrijk, Belgium; 2 Institute for Biostatistics and statistical Bioinformatics (I-BioStat), Hasselt University, Diepenbeek, Belgium; 3 School of Public Health and Community Medicine, The University of New South Wales, Sydney, Australia

**Keywords:** VAS, EQ-5D, Alcohol, Gender, Smoking, Pet

## Abstract

**Purpose:**

To identify determinants of health-related quality-of-life in the Belgian population and to provide age-specific population norms of health-related quality-of-life.

**Methods:**

Between September 2010 and February 2011, a representative sample of 1774 persons (age 0–99) was surveyed using the standard Euroqol questionnaire (EQ-5D-3L) with a Visual Analogue Scale (VAS). Significant determinants were identified using multivariate beta (VAS) and one-inflated beta (EQ-5D) regression, the latter modelling the probability to be in perfect health separately from the average EQ-5D score if not in perfect health.

**Results:**

Health-related quality-of-life depends largely on age and experience with severe disease. The probability to be in perfect health is highest for children. For 0–2 years children who are not in perfect health, proxies report EQ-5D and VAS scores as low as that of the elderly. Also smoking behaviour, educational attainment, pet ownership, working or having worked in health care, and potentially household size and 60+ living on their own (yes/no) are associated with health-related quality-of-life, whereas no association was found with gender, living in a single-parent home, educational attainment of mothers, alcohol consumption of 60+, having (grand-) children and the frequency of seeing them. The same determinants are significant for VAS and the probability to be in perfect health, but not for the average EQ-5D score if not in perfect health.

**Conclusions:**

The population norms provided can be used directly as input in health economic evaluations. Estimating health-related quality-of-life in children and developing statistical tools capturing the particular features of health-related quality-of-life measures are important areas for future research.

**Electronic supplementary material:**

The online version of this article (doi:10.1007/s11136-017-1556-y) contains supplementary material, which is available to authorized users.

## Introduction

Baseline health-related quality-of-life (HRQoL) values, sometimes referred to as ‘population norms’ or ‘population reference data’, have been presented for several countries e.g. [1, 2], but usually—as for Belgium—only for a fixed set of relatively wide age groups. Hence differences in HRQoL between members of the same age group are not quantified. Furthermore, in order to improve public health, it is crucial to understand which socio-demographic and economic characteristics are associated with poor health. An acceptable ratio of costs to Quality-Adjusted Life-Years (QALYs, based on the standard Euroqol questionnaire EQ-5D) is a requirement for new health care interventions to receive government funding in many European countries (including Belgium). Therefore it is of major interest to understand better what influences the production of QALYs in the population. Older age, being a woman, having lower educational attainment, lower income and being a smoker have been shown to be associated with poorer HRQoL [1, 3–8]. However, all studies showing such associations focused on a subgroup of the general population (e.g. university students, elderly) [3–8]. Also, the impact of alcohol consumption and pet ownership has been investigated to a lesser extent [9–12] and the impact of various other characteristics such as occupation, household size, living in an elderly home and frequency with which elderly are visited by their children and grandchildren has not been investigated at all. Insights in additional determinants of health could directly impact best practice of applied cost-utility analyses, for instance for interventions targeted at health care workers or elderly. General age-specific population norms may not apply to these groups to assess the impact of a specific disease on their quality of life evaluation.

We aim to identify significant determinants of HRQoL in the Belgian population. Rather than exploring a few determinants of choice, we opt here to let the data reveal influential determinants out of an extensive available set of 34 plausible determinants, of which many have not been studied before. Additionally, we aim to provide HRQoL population norms for Belgium as a continuous function of age in years. We use (one-inflated) beta regression analysis [13], a statistical technique that captures the specific features of the HRQoL score: constrained between a minimum score for worst health and a maximum score for perfect health, and—when measured in the general population—having a skewed distribution with most people being healthy.

## Methods

### Survey

We surveyed HRQoL in the general population in Flanders (60% of the Belgian population) between September 2010 and February 2011, using the standard Euroqol questionnaire (EQ-5D-3L) with a Visual Analogue Scale (VAS). One person per household was recruited by random digit dialling on mobile phones and landlines. Quota sampling by age, gender and region was applied in order to achieve a representative sample. People who agreed verbally to participate were sent a written questionnaire by mail with a pre-stamped envelope to return the completed questionnaire.

Three types of questionnaires were used, adapted to the ages of the participants. For example, the questionnaires for children (0–12 years) included questions on school and education of the mother, and were completed by a proxy (parent). Although adult valuations of health were not intended to be used in children, we do consider them, as HRQoL estimates are often demanded for health economic evaluations (in particular cost-utility analyses) for childhood interventions. We believe it is better to estimate population norms by age than assuming the average child is in perfect health, which is often current practice. However, since the HRQoL outcomes have not been validated for children, we perform all analyses with and without the inclusion of the child-by-proxy questionnaires. Questionnaires for the elderly (>60 years) included questions on whether they were living in an elderly home, and contained instructions for proxies to help the participants. Table 1 gives an overview of all requested information by age group.

**Table 1:** Overview of the health-related quality-of-life measures and the 34 potential determinants measured in the survey, with the percentage of missingness in each variable

All age groups (*n* = 1774)	Categories or [range]	% missing
EQ-5D dimension 1	No problems with walking around; some problems with walking around; confined to bed	1
EQ-5D dimension 2	No problems with caring for myself; some problems with caring for myself/washing or dressing myself; not able to wash or dress myself	1
EQ-5D dimension 3	No problems with daily activities; some problems with daily activities; not able to do daily activities	1
EQ-5D dimension 4	No pain or other complaints; moderate pain or other complaints; severe pain or other complaints	1
EQ-5D dimension 5	Not anxious or depressive; moderate anxious or depressive; very anxious or depressive	1
EQ-5D index score	[−0.074,1]	2
VAS score	[0, 100]	5
Age	[0, 99]	0
Gender	46% male; 54% female	0
Household size	[1, 12]	2
Experience with severe disease with oneself	16% yes; 77% no	7
Experience with severe disease with family member	48% yes; 41% no	10
Number of parents	23% 1; 72% 2	5
Animals at home	59% yes; 40% no	1
Normal day	75% yes; 24% no	0
Children (0–12 years, *n* = 317)		
Education level of the mother	1% primary school; 10% vocational; 2% lower technical; 3% lower secondary; 5% higher technical; 13% higher secondary; 44% higher non-university; 21% university or post-university	1
Reason not a normal day child (*n* = 96)	11% sick; 20% daycare closed; 66% other	3
Often in daycare?	54% yes; 45% no	1
Number of other children in daycare	[0, 400]	3
School (yes/no + type)	29% kindergarten; 40% primary school; 5% other school; 25% no	1
Number of children in school	[6, 30]	3
Adults (13–65 years, *n* = 1095)		
Smoking behaviour adult	64% no smoker; 19% ex-smoker; 17% smoker	0
Adults and elderly (*n* = 1457)		
Education level	1% none; 7% primary school; 10% vocational; 5% lower technical; 5% lower secondary; 10% higher technical; 13% higher secondary; 27% higher non-university; 12% university or post-university; 9% student	1
Reason not a normal day adult and elderly (*n* = 343)	10% sick; 15% holiday; 1% care for a sick family member; 71% other	3
Experience with severe disease because caring for someone else	8% yes; 62% no	30
Work(ed) in health sector	22% yes; 77% no	1
Profession	4% craftsman, employer without employees; 1% craftsman, employer with 5 or less employees; 1% craftsman, employer with 6 or more employees; 3% professional services; 3% senior member of general management; 13% middle, not part of the general management; 33% other employee; 6% worker with vocational training; 8% worker without vocational training; 7% housewife or househusband; 2% disabled; 2% retired; 9% student; 3% unemployed; 0.1% rentier; 5% more than 1 profession	1
Elderly (60–99 years, *n* = 362)		
Smoking behaviour elderly	12% smoker; 8% quit smoking before age 30; 16% quit smoking between age 30 and 50; 13% quit smoking after age 50; 48% never smoker	3
Working or retired	6% working; 92% retired	1
Frequency seeing children (*n* = 324)	17% daily; 42% several times a week; 28% several times a month; 2% once a month; 5% several times a year; 1% once a year; 2% less than once a year	2
Frequency seeing grandchildren (*n* = 306)	8% daily; 32% several times a week; 31% several times a month; 9% once a month; 11% several times a year; 2% once a year; 3% less than once a year	5
Frequency drinking alcohol (*n* = 257)	23% daily 1 or 2 glasses; 7% daily more than 2 glasses; 25% a couple of times a week 1 or 2 glasses; 7% a couple of times a week more than 2 glasses; 23% a couple of times a month; 12% a couple of times a year	4
Where do you live?	86% house, apartment or service flat; 2% elderly home; 11% elderly and caring home; 1% with family	1
If living in elderly home, with how many people? (*n* = 46)	4% 10–30; 9% 30–50; 37% 50–100; 48% >100	7
Drinking alcohol and if yes, what type	14% yes, mostly beer; 33% yes, mostly wine; 19% yes, as much beer as wine; 3% yes, mostly liquor; 29% no	2
Do you have children?	89% yes; 10% no	1
Number of children	[0, 12]	2
Age youngest child	[13, 74]	5
Do you have grandchildren?	82% yes; 15% no	3
Number of grandchildren	[0, 36]	4
Age youngest grandchild	[0, 56]	6

Data were single-entered in an electronic database and double-checked manually. No physical samples were collected as part of this study and the ethical committee of the Antwerp University Hospital approved the study protocol. Participants were able to refuse participation even after verbal agreement by not filling in the questionnaire, and/or by not sending it back. The first page of the questionnaire explained that their answers would be used anonymously for scientific research purposes at our universities. Thus, the fact that they filled in the questionnaire and chose to send it in functions as a written consent. We obtained similar verbal consent with implicit written confirmation from the next of kin, caretakers or guardians on behalf of dependent participants (e.g. children).

In total, 1774 of the 2760 approached individuals completed a questionnaire (i.e. response rate of 64%). This included 18% children below 13 years and 20% adults older than 60 years. The data can be found in the Electronic Supplementary Materials (Survey Data).

### Analysis

A single EQ-5D index score per respondent was calculated by applying the scoring algorithm for Belgium to the levels of the 5 separate dimensions [14]. The survey data had several complexities, including missing values, right-skewed distributed HRQoL scores confined between 0 and 1 (VAS) or between −0.074 and 1 (EQ-5D index score), variables only measured for children, adults and/or elderly and categorical variables with a large number of categories. The analytical procedure to deal with these complexities is detailed in the following paragraphs. All analyses are done in R version 3.0.2 [15].

#### Imputing missing values

Table 1 shows the percentage of missing values for the HRQoL measures and each of the 34 covariates. Fully conditional specification was used to impute the missing values (sometimes called ‘sequential regression multiple imputation’, R package ‘mice’ [16]). By default, a burn-in period of 5 iterations was used (sensitivity analysis using 5, 20, 50 and 200 burn-in iterations showed convergence of all variables to be imputed). Each variable with missing values was regressed on all other variables in the dataset with the regression model depending on the type of variable: logistic regression for binary variables, polytomous logistic regression for factors with more than 2 levels, predictive mean matching for non-normally distributed continuous variables [17] and a classification tree for the variable ‘having animals (yes/no)’ (using logistic regression to impute this variable for the children resulted in imputation errors). Categorical covariates only measured in a subgroup of the sample (e.g. smoke status for adults and elderly) were assigned the category ‘irrelevant’ for the subgroup in which they were not measured (e.g. smoke status for children) so that they were not imputed. Continuous covariates only measured in a subgroup of the sample (e.g. age of the youngest grandchild is only measured for elderly having grandchildren) were imputed but removed during a post-processing procedure, and were not used as predictors to impute other covariates [18]. To explore the uncertainty in the results due to imputation, all analyses were done on five imputed datasets after which results were merged.

#### (One-inflated) beta regression analysis for identifying significant determinants

Beta regression (R package ‘gamlss’ [19]) was used to model VAS as a function of covariates as it captures its specific features: constrained between a minimum score for worst health and a maximum score for perfect health, and—when measured in the general population—having a skewed distribution with most people being healthy [13]. One-inflated beta regression (R package ‘gamlss’ [19]) was used to model the EQ-5D index score, as a large part of respondents had a score of ‘1’ (perfect health) [13]. That is, not only the probability to be in perfect health is modelled as a function of important covariates (‘nu model’), but also the average EQ-5D index score if not in perfect health (‘mu model’).

##### Transforming outcome variables: VAS and EQ-5D index score

As the beta distribution is defined between (i.e. excluding) 0 and 1, the original VAS variable (range 0–100) was divided by 100 and shrunken so that it fell between (excluding) 0 and 1 [20]:

1 $${\text{VASne}}{{\text{w}}_{\text{i}}}{\text{ = }}\left( {{\text{VA}}{{\text{S}}_{\text{i}}} \times \left( {{\text{N}} - {\text{1}}} \right){\text{ + 0}}{\text{.5}}} \right){\text{/N, for i = 1, }}...{\text{, N respondents and N = sample size}}$$

Because the EQ-5D index score can be negative (which was the case for one respondent in our sample), and the maximum non-one value is 0.817, the non-one values were first normalized so that they fell between (including) 0 and 1:

2 $${\text{EQ-5Dnorm}}_{{\text{i}}} {\text{ = }}\left[ {{\text{EQ-5D}}_{{\text{i}}} - {\text{min}}\left( {{\text{EQ-5D}}} \right)} \right]{\text{/}}\left[ {{\text{max}}\left( {{\text{EQ-5D}}} \right) - {\text{min}}\left( {{\text{EQ-5D}}} \right)} \right]$$

The normalized non-one values were then transformed using the same formula (1) as for VAS.

##### Defining determinants

All 34 potential determinants included in the analysis are shown in Table 1. Seventy-one participants indicated more than one profession (5%, see Table 1). The two participants that specified both ‘disabled’ and another profession were classified as ‘disabled’. The other 69 participants were grouped in a separate category ‘more than one profession’. Indicators (yes/no variables) were used for the variables measured only in children, adults and/or elderly, i.e. for these variables the indicator variable as main effect (e.g. children (1 = yes, 0 = no)) and the interaction effect of the variable and indicator was included (e.g. children (1 = yes, 0 = no) × school). In exploratory analysis polynomials, fractional polynomials and cubic splines were used to model the health score as a function of age and household size. These fitted the data almost equally well and therefore second order polynomials were considered for household size and age.

##### Model selection

Model selection was done in two steps. In a first step, backward elimination was done manually by removing all covariates that were not significant (p > 0.05) in the regression models for any of the five imputed datasets. Ideally, backward elimination should have been applied on the pooled results of the five imputed datasets. We considered this too laborious given the expected limited added value. Indeed, much time would go into bootstrapping to obtain reliable variance–covariance estimates for pooling results (see below 2.3.5) and into handling the lack of an automatic procedure for backward elimination on these pooled results. No dramatic changes in regression coefficients were observed during the backward selection process (i.e. no strong multicollinearity).

In a second step, variables with a large number of categories (e.g. 10 education levels) were redefined to enable interpretation of variables. Recategorization was based on subject knowledge and complemented with statistical tests. For example, if the education variable was significant, but the HRQoL of only one of the ten educational categories was significantly different from the others, education was redefined as having two categories instead of ten if these categories were meaningful. Models with the original and the redefined variable were compared with AIC (Akaike, 1973), and the variable definition resulting in the model with the lowest AIC value was retained. For each variable, this was done for all possible reference categories to make sure category reduction was independent of the reference category. As such, observed differences can potentially be overinterpreted, but this approach was preferred over the more subjective approach of manually reducing the number of categories prior to the analysis based on what is thought to represent ‘sensible’ categories.

##### Diagnostics

Diagnostic plots (QQ plots and plots of randomized quantile residuals against age) and tests (Cox-Snell pseudo R^2^) were used to assess the goodness-of-fit of the final models as well as to identify possible influential values (outliers).

##### Pooling results

Because no reliable variance–covariance matrices could be obtained using the gamlss function in R, we used bootstrapping to obtain pooled regression coefficient estimates and pooled variances of these estimates (*n* = 500, this rendered stable results). This is, we fitted 500 × 5 regression models (on the 500 datasets bootstrapped from each of the five imputed datasets) and obtained the regression coefficients for each imputed dataset by taking the mean of the 500 estimated regression coefficients from the model fitted on each imputed dataset. The variance–covariance matrix of the regression coefficients for each imputed dataset was obtained by taking the variance and covariance over all 500 estimated regression coefficients for each imputed dataset. Next, Rubin’s rules [21] were applied to obtain the pooled regression coefficients, and the within- and between-imputation variance, as well as the total variance and the proportion of variation due to imputation (lambda).

##### Sensitivity analysis

To account for the fact that adult health valuations were not intended to be used in children, all analyses were repeated using the data from the adults and elderly only.

#### Population norms

Beta and one-inflated beta regression models were fitted with the VAS and EQ-5D index score, respectively, as an outcome and age as the only explanatory variable. Confidence regions around the estimates were obtained by bootstrapping (500 replicates, this rendered stable results). Analyses were performed with and without including the data for children.

## Results

### Determinants of health-related quality-of-life

Final regression models are shown in Table 2 (VAS score) and 3 (EQ-5D index score). The Cox-Snell pseudo R^2^ for the final models are 0.28 for the EQ-5D index score as outcome and 0.31 for the VAS score as outcome. QQ plots and plots of randomized quantile residuals against age (Figs S5 and S6 in Electronic Supplementary Material) indicate a relative good fit of the beta distribution for the model with VAS as an outcome, but a less good fit of the one-inflated beta distribution for the model with the EQ-5D index score as outcome. One outlying EQ-5D index score and two outlying VAS scores were identified. Analyses excluding these outlying records revealed similar results as when including them (based on the analyses including all determinants and including the data for children), therefore these outlying records were retained.

**Table 2:** Pooled estimates for the final beta regression model for VAS score including 10 determinants

	Pooled estimate	ll	ul	Within-imputation variance	Between-imputation variance	Total variance	Proportion variation due to non-response (%)
Intercept	1.25	0.80	1.69	0.05	0.001	0.05	2
Age	0.03	0.01	0.04	0.0001	0-0.0001	0.0001	9
Age squared	−0.0003	−0.0005	−0.0001	0 to 0.0001	0 to 0.0001	0 to 0.0001	15
Experience severe disease with oneself	−0.45	−0.57	−0.31	0.004	0.0002	0.005	5
Experience severe disease with family member	−0.20	−0.32	−0.09	0.003	0.0005	0.003	16
Pets	−0.16	−0.26	−0.05	0.003	0.0002	0.003	10
Household size	0.03	−0.01	0.08	0.0004	0.0001	0.0005	22
Child	−0.07	−1.05	1.50	0.92	0.004	0.92	0
Sick	−0.87	−1.11	−0.62	0.02	0.0009	0.02	7
Child*sick	−1.12	−1.51	−0.73	0.04	0.002	0.04	6
Disabled	−0.79	−1.03	−0.55	0.01	0.0006	0.01	5
Work(ed) in health sector	0.16	0.03	0.29	0.004	0.0005	0.005	12
Education level: none or primary school	−0.19	−0.36	−0.01	0.008	0.001	0.010	13
Education level: higher (not-) university	0.02	−0.10	0.14	0.003	0.0001	0.004	4
Education level: student	0.36	0.05	0.67	0.02	0.001	0.03	6
Elderly	0.10	−0.13	0.32	0.01	0.0009	0.01	8
Living in elderly home or with family	−0.33	−0.68	0.08	0.02	0.01	0.04	33

**Fig. 1:**
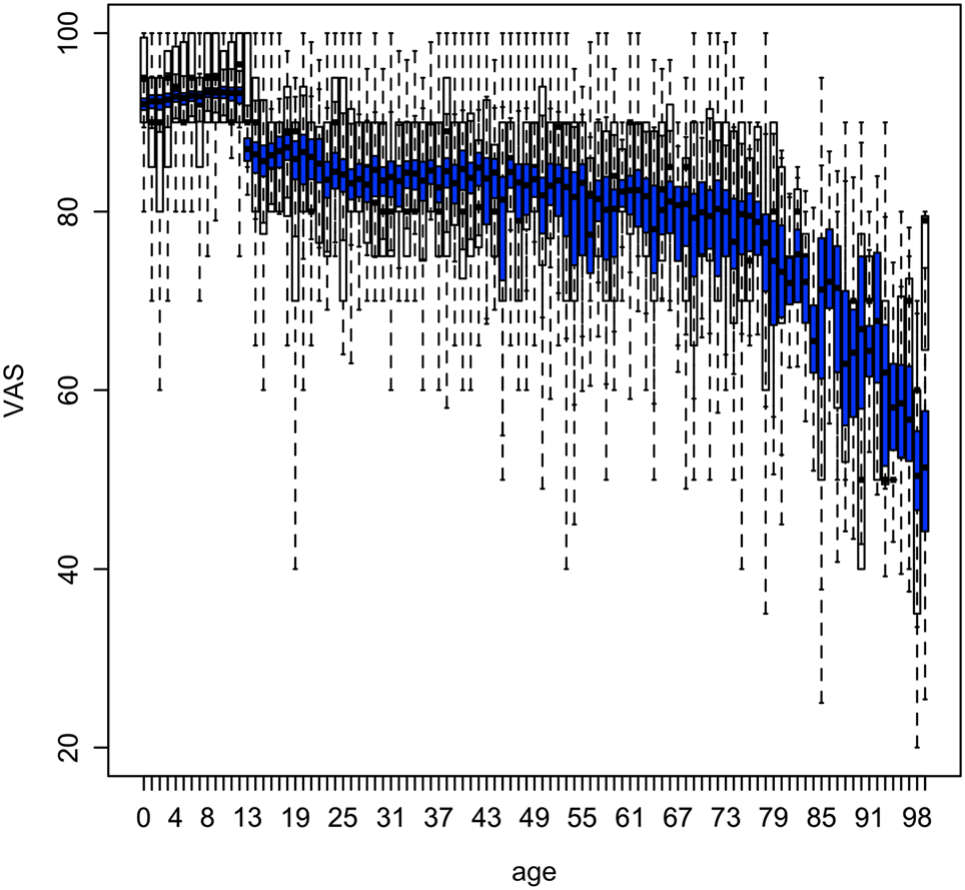
VAS score as a function of age in years, based on a beta regression model including 10 determinants. *Boxplots* of observed and predicted (*blue*) VAS scores on their original scale. *Boxplots* of predicted VAS scores represent within- and between-imputation variance (see Table 2)

**Fig. 2:**
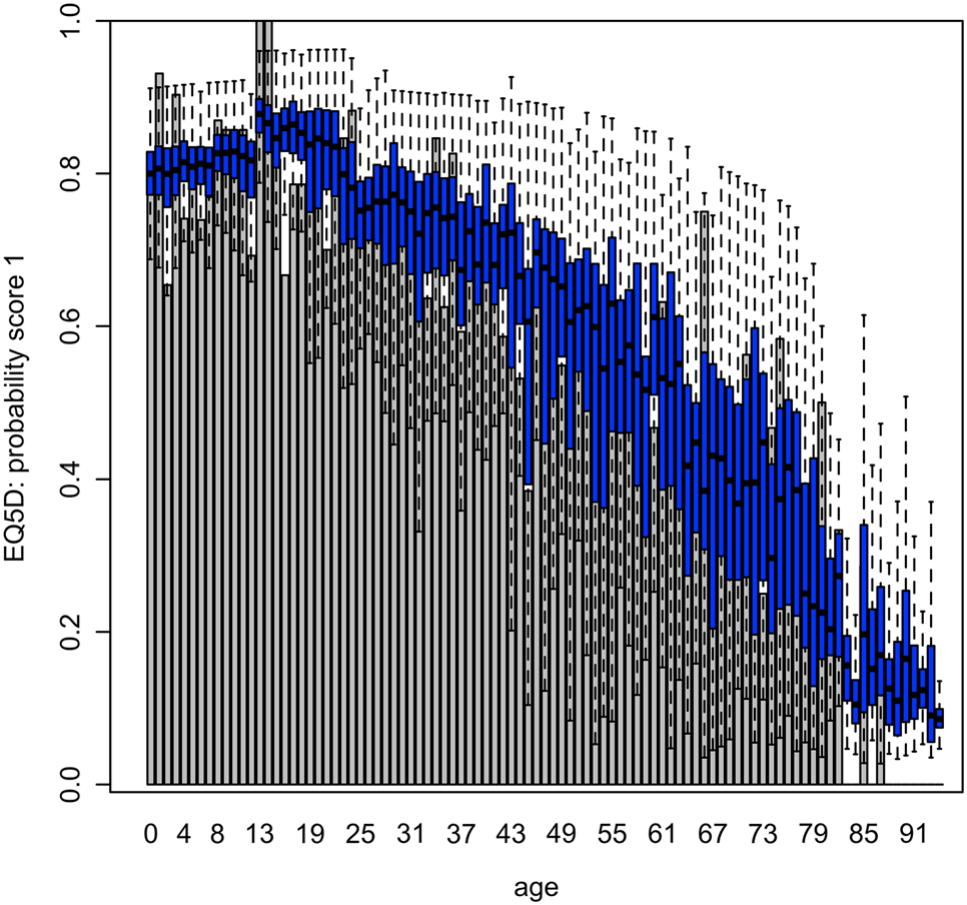
Probability to be in perfect health (EQ-5D index score of 1) as a function of age in years, based on a one-inflated regression model including 7 determinants. *Bars* show observed probabilities. *Boxplots* show predicted probabilities on their original scale, representing within- and between-imputation variance (see Table 3, nu model)

**Fig. 3:**
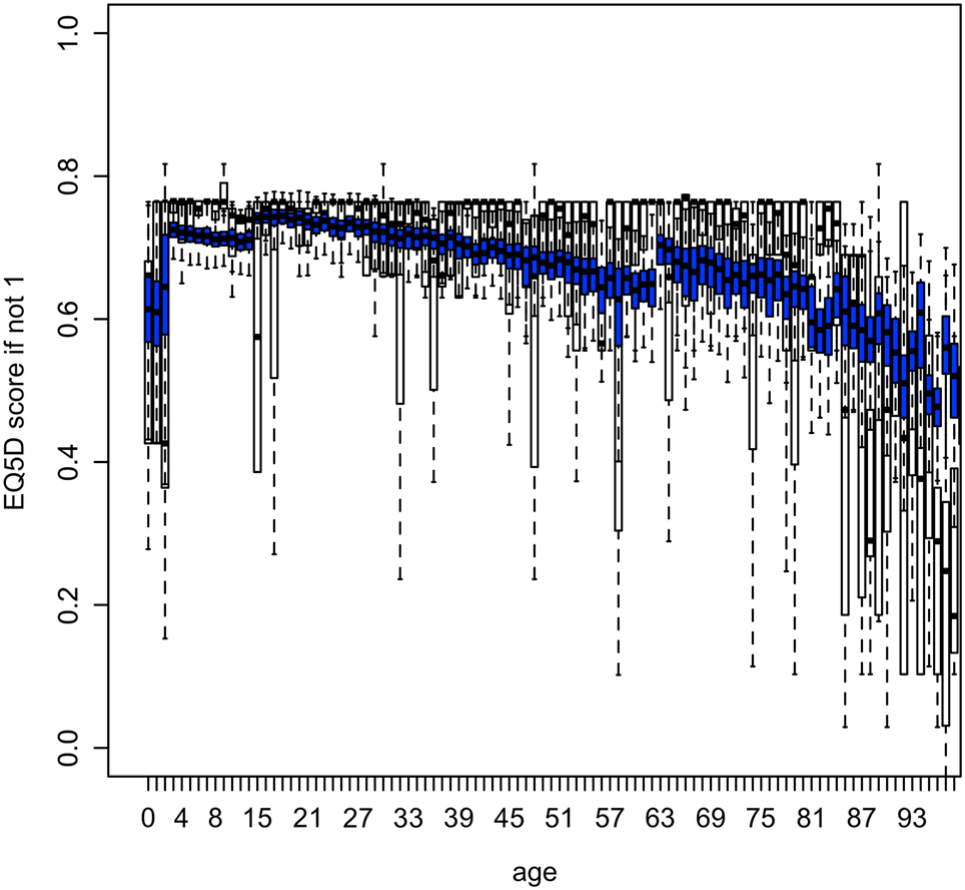
EQ-5D if not in perfect health (EQ-5D index score < 1) as a function of age in years, based on a one-inflated regression model including 10 determinants. *Boxplots* of observed and predicted (*blue*) EQ-5D scores (if not 1), on their original scale. *Boxplots* of predicted scores represent within- and between-imputation variance (see Table 3, mu model)

Estimated HRQoL (original scale) based on the final regression models as a function of significant determinants is presented in Figs. 1, 2 and 3 as a function of age averaged over all other determinants, and in Table 4, Figs S1 and S2 (Electronic Supplementary Material) as a function of all significant determinants except for age.

**Table 3:** Pooled estimates for the final one-inflated beta regression model for EQ-5D index score including 13 determinants

	Pooled estimate	ll	ul	Within-imputation variance	Between-imputation variance	Total variance	Proportion variation due to non-response (%)
*Nu model for the probability to be in perfect health (i.e. EQ-5D index score = 1)*							
Intercept	−10.13	−18.32	2.04	80.50	0.05	80.57	0.08
Age	0.04	0.006	0.08	0.0003	0 to 0.0001	0.0003	2
Age squared	−0.0007	−0.001	−0.0004	0-0.0001	0 to 0.0001	0 to 0.0001	2
Sick	−1.66	−2.44	−0.95	0.12	0.01	0.14	12
Experience severe disease with oneself	−1.03	−1.33	−0.73	0.02	0.001	0.02	5
Experience severe disease with family member	−0.45	−0.69	−0.21	0.01	0.0008	0.02	6
Pets	−0.37	−0.61	−0.13	0.02	0.0001	0.02	1
Adult or elder	11.03	−1.48	19.06	80.57	0.082	80.67	0.1
Education level: none or primary school	−0.90	−1.45	−0.39	0.07	0.001	0.07	2
Education level: higher (not-) university	0.42	0.15	0.69	0.02	0 to 0.0001	0.02	0.5
Education level: student	1.14	0.47	1.90	0.13	0.002	0.13	2
Work(ed) in health sector	−0.36	−0.64	−0.07	0.02	0.0001	0.02	0.8
*Mu model for the average EQ-5D score if not in perfect health*							
Intercept	2.63	1.95	3.32	0.11	0.02	0.14	18
Age	−0.02	−0.03	−0.01	0 to 0.0001	0 to 0.0001	0 to 0.0001	4
Experience severe disease with oneself	−0.22	−0.39	−0.04	0.008	0.0003	0.008	5
Experience severe disease with family member	0.25	0.04	0.46	0.009	0.002	0.01	25
Household size	0.17	0.02	0.38	0.006	0.0006	0.007	10
Household size squared	−0.02	−0.06	−0.006	0.0001	0 to 0.0001	0.0001	9
Child	−0.96	−1.56	−0.32	0.10	0.004	0.11	5
Not going to (regular) school	−0.83	−1.72	0.10	0.22	0.007	0.23	4
Adult	−0.52	−0.82	−0.24	0.02	0.0006	0.02	3
Disabled	−0.91	−1.24	−0.57	0.03	0.0001	0.03	0.4
Drink no or rarely alcohol	−0.43	−0.72	−0.16	0.02	0.0009	0.02	5
Smoking	−0.27	−0.49	−0.07	0.01	0.0002	0.01	3
No experience severe disease because of caring for someone	0.17	−0.07	0.41	0.01	0.003	0.02	21
Work(ed) in health sector	0.16	−0.008	0.34	0.006	0.002	0.008	23

Older age and experience with severe disease were significantly associated with a lower HRQoL in all analyses (Tables 2, 3). The VAS score was estimated to be substantially higher for children (90–95) than for adults and elderly (<90), decreasing slowly to 80 for age 75 and more rapidly thereafter (Fig. 1). The probability to be in perfect health increased from 80% in newborns to almost 90% for 15-year olds and decreased thereafter rapidly to less than 10% for persons age 80 and older (Fig. 2). For persons not in perfect health, EQ-5D decreased with increasing age, with a different intercept for children, adults and elderly (Fig. 3; Table 3), but was especially low for children aged 0–2 years (0.43). The latter was captured in our model by the variable ‘no school’ (although not significant based on pooled confidence interval, Table 3), as in Belgium children start going to school at the age of 2.5–3 years.

Being sick or disabled, having lower educational attainment, owning pets and smoking (adults) were significantly associated with a lower HRQoL for all 5 imputed datasets, but not for all HRQoL outcome measures. Notably, the determinants retained for VAS and the probability to be in perfect health were similar, but different from the ones retained for the average EQ-5D score for persons not in perfect health.

Being sick was based on merging the variables ‘normal day’ and ‘reason not a normal day’ for children, adults and elderly. Persons being sick at the moment the questionnaire was completed had a lower probability to be in perfect health and report a much lower VAS score than healthy persons. The impact of being sick on VAS score was larger for children (score 63 versus 93) than for adults and elderly (68 versus 83) (i.e. significant interaction effect). Persons indicating under ‘profession’ that they were disabled had significantly lower VAS and EQ-5D scores when not in perfect health than persons who filled in any of the other options under ‘profession’. The covariate education level could be reduced to four levels (no or primary school, secondary school, higher (non-)university education and students). HRQoL increased with increasing education level, with students having the highest probability to be in perfect health and the highest VAS score. Education was only for a part of the imputed datasets a significant determinant for VAS. If corrected for age, persons owning pets reported lower VAS scores and a lower probability to be in perfect health. This effect was not observed in Table 4, as in our survey pet owners were on average 10 years younger than persons without a pet (age 37 compared to 47 years) and younger age was associated with a better HRQoL. Adults not in perfect health had a lower EQ-5D score if they were current smokers than if they did not smoke (anymore).

**Table 4:** Observed and fitted health-related quality-of-life scores and probabilities as a function of significant categorical variables

		VAS		EQ-5D probability perfect health		EQ-5D if not in perfect health	
		Data	Model	Data	Model	Data	Model
Experience severe disease with oneself	No	85	85 [68–94]	0.71	0.70 [0.20–0.91]	0.69	0.69 [0.55–0.75]
	Yes	72	72 [49–89]	0.34	0.33 [0.03–0.71]	0.58	0.62 [0.42–0.72]
Experience severe disease with family member	No	86	86 [63–94]	0.75	0.73 [0.15–0.92]	0.65	0.67 [0.49–0.74]
	Yes	80	79 [56–92]	0.57	0.55 [0.06–0.86]	0.67	0.68 [0.51–0.75]
Experience severe disease because of caring for someone	No					0.67	0.68 [0.52–0.75]
	Yes					0.63	0.64 [0.46–0.74]
Going to (regular) school	No					0.51	0.60 [0.47–0.71]
	Yes					0.74	0.72 [0.67–0.75]
Smoking	No					0.70	0.70 [0.50–0.75]
	Yes					0.61	0.65 [0.67–0.73]
Drinking alcohol	No or rarely					0.66	0.69 [0.54–0.75]
	Yes					0.64	0.67 [0.56–0.73]
Disabled	No	81	81 [61–89]			0.66	0.68 [0.53–0.75]
	Yes	61	60 [40–74]			0.47	0.51 [0.34–0.63]
Work(ed) in health sector	No	80	80 [56–88]	0.61	0.60 [0.06–0.91]	0.64	0.67 [0.49–0.75]
	Yes	82	82 [61–90]	0.57	0.56 [0.07–0.85]	0.69	0.69 [0.53–0.75]
Highest education level	None or primary	70	69 [44–85]	0.23	0.22 [0.01–0.62]		
	Secondary	80	80 [57–88]	0.54	0.53 [0.09–0.80]		
	Higher education	83	82 [68–89]	0.69	0.68 [0.25–0.88]		
	Student	85	86 [76–91]	0.86	0.86 [0.64–0.95]		
Pets	No	82	82 [55–94]	0.62	0.62 [0.04–0.91]		
	Yes	83	83 [62–94]	0.65	0.64 [0.15–0.89]		
Sick	No	83	83 [60–94]	0.64	0.64 [0.09–0.90]		
	Yes	65	64 [37–77]	0.32	0.34 [0.01–0.70]		
Living in elderly home or with family	No	78	77 [61–86]				
	Yes	61	60 [43–76]				

Mean observed scores (medians and quartiles are presented in Figs S1-S2 in the Electronic Supplementary Material). Fitted means with 95% confidence intervals reflecting both within- and between-imputation variance. Fitted values for VAS and EQ-5D are given on their original scale

Having experience with severe disease of a family member was associated with a lower probability to be in perfect health and a lower VAS score, but a slightly higher (see below) EQ-5D index score if not in perfect health. Working/having worked in the health care sector was associated with a lower probability to be in perfect health, but on average a higher VAS score and EQ-5D index score if not in perfect health. For the latter score, this effect was significant for only 2 of the 5 imputed datasets and not significant based on pooled confidence interval (Table 3). Adults not in perfect health had a lower EQ-5D score when they rarely or never drank alcohol. This effect was however driven by the one person with a negative EQ-5D score: when excluding this extreme low EQ-5D score from the analysis, the effect was not significant anymore.

For the following variables, the effect was significant for only 2 of the 5 imputed datasets and not significant based on the pooled confidence interval (Tables 2, 3). The larger the household size, the higher the VAS score. Persons living in households of size 5 had highest EQ-5D score if not in perfect health, with persons living in smaller or larger households reporting lower EQ-5D scores. Elderly had a lower VAS score when living in a nursing home or with family than when living in a house or (service) flat. Adults having experience with severe disease by taking care of someone else had a lower EQ-5D score if not in perfect health (not significant based on pooled confidence interval, Table 3). Note that for this covariate 30% of the responses were missing (Table 1).

Gender and number of parents were not associated with HRQoL in any of the analyses. Specifically for children no association was found with educational attainment of the mother, nor with whether they regularly attend daycare and the size of the daycare centres or school classes. For the elderly, no association was found with the smoking behaviour, being retired, alcohol consumption (volume and type), the size of the home for the elderly, having children or grandchildren, the frequency with which children and grandchildren are visiting, the number of children and grandchildren and the age of the youngest child and grandchild.

Similar results are obtained when excluding the data for children (see S1 Text in Electronic Supplementary Material), with a number of notable exceptions. The living status of the elderly (on their own/in an elderly home or with family) became important in determining the EQ-5D index score if not in perfect health, when using the data of adults and elderly only (S1 Text Table 2). In contrast, household size for VAS and age, school and alcohol consumption for the EQ-5D score if not in perfect health were not retained in the final regression models (S1 Text Tables 1, 2). Pooled estimates of the regression coefficients and fitted values were very similar with (Tables 2, 3, 4) or without (S1 Text Tables 1, 2, 3) including the data for children, the only exception being the variable ‘sick (yes/no)’. This is because the proportion stating perfect health despite being sick was higher in children (five out of nine) than in adults (nine out of thirty-five). The confidence intervals around the fitted values based on the data of adults and elderly only (S1 Text Table 3) were slightly wider than (if not similar to) the confidence intervals around the fitted values based on the data for all age groups (Table 4). Only for the fitted VAS scores as a function of having had experience with severe disease or having had pets, the confidence intervals are slightly narrower when excluding the data for children. This is because the difference in VAS score for these predictors is slightly less pronounced for children than for adults and elderly (e.g. VAS score for children having experienced severe disease is 81 ± 20 (mean ± SD) versus 92 ± 9 without such experience, whereas the VAS score for adult and elderly having experienced severe disease with a family member is 71 ± 17 versus 83 ± 11 without such experience).

### Population norms Belgium: health-related quality-of-life as a function of age

Final regression models are shown in S1 Table (Electronic Supplementary Material). The Cox-Snell pseudo R^2^ for the final model with EQ-5D index score as outcome is 0.18, and 0.16 for the final model with VAS as outcome. QQ plots (Fig S5 in Electronic Supplementary Material) indicate a relatively good fit of the beta distribution for the model with VAS as an outcome, but a less good fit for the model with the EQ-5D index score as an outcome. Plots of randomized quantile residuals as a function of age (Fig S6 in Electronic Supplementary Material) show a clear pattern, indicating not a good model fit. Therefore, we present as population norms not only the estimates based on the models including age only, but also based on the models including all significant determinants (S2 Table in Electronic Supplementary Material). Although the models including significant determinants do not estimate HRQoL as a continuous function of age (due to the use of indicator variables to handle covariates not measured in all age groups), they fit the data well (see first paragraph of the “Results” section). This is further discussed in the “Discussion” section.

S3 Fig (VAS score) and S4 Fig (EQ-5D index score) show the population norms for Belgium based on the models only including age as a covariate. Estimated average VAS and EQ-5D index scores by age in years and with 95% confidence intervals are presented in S2 Table (Electronic Supplementary Material). The average VAS score is estimated to be around 84 for teenagers and to decrease down to 81 for the people aged 60 years. The model including all significant determinants estimates the VAS score to be around 90 for children (<13 years of age) and to decrease down to 58 for the 95 year olds. The model including only age as a covariate underestimates the average score of children and overestimates the average score of people aged 60 years or more. The average EQ-5D index scores based on the model including only age are very similar to the ones estimated by the model including all significant determinants (S2 Table, Electronic Supplementary Material). The average EQ-5D index score is estimated to be around 0.94 for children and to decrease down to less than 0.60 for the very old (89+). Both models underestimate slightly the scores for the 67–77 years olds and overestimate slightly the scores for the 87+.

Similar EQ-5D index scores by age are obtained when excluding the data from children (see S1 Text in Electronic Supplementary Material). The estimated VAS scores by age based on the data from adults and elderly only are lower for the older age groups (S1 Text Table 5). Also, the between-imputation variance of the regression coefficients for the VAS models based on the data from adults and elderly only (S1 Text Table 4) is larger than the between-imputation variance for the VAS models based on the data from all age groups (S1 Table, Electronic Supplementary Material). This is because the variation in imputed VAS scores is larger for adults and elderly than for children.

## Discussion

HRQoL in adults (measured with EQ-5D) has been shown to decrease with increasing age [6, 22]. One-inflated beta regression revealed that the probability to be in perfect health decreases much faster than the average EQ-5D score if not in perfect health. Additionally, teenagers showed the highest VAS score and probability to be in perfect health, whereas proxies reported low HRQoL scores for children aged 0–2 years and not in perfect health. Hence, unlike what is usually assumed in health economic evaluations, children in the general population are not evaluated by their proxies to be in perfect health. These results should however be interpreted with care, as it remains unclear to which extent proxies are able to provide objective and consistent information on the HRQoL of children [23].

The negative association between HRQoL and education level, smoking, and living in a nursing home has been documented before, but not for EQ-5D and VAS ([3, 5–8, 24–28].

The significance of pet ownership and alcohol consumption [9, 10, 29] for HRQoL is inconclusive between studies. We found a negative association between pet ownership and the average HRQoL, whereas Lewis et al [11] found a positive association and Maynard [12] found no association, but Lewis et al [11] and Maynard [12] studied only university students. The three studies used different measures for HRQoL and ‘pet ownership’ was defined differently. We studied alcohol consumption only in elderly and found a significant effect of the frequency with which alcohol is consumed, but this was due to a single person with an extremely low HRQoL score.

Persons witnessing severe disease in family members have a lower probability to be in perfect health and report a lower VAS score. This may be related to the significant psychological and physical burden of caring for an ill family member [30, 31]. On the contrary, we found that these persons, as well as persons who had experience with severe disease by taking care of someone else, have a slightly higher EQ-5D score if not in perfect health, than persons without such experience. Also working/having worked in the health care sector was found to be associated with a lower probability to be in perfect health, but on average a higher VAS score and EQ-5D index score if not in perfect health. To understand these seemingly contrasting results better, it may be useful to look at how these determinants influence each of the 5 dimensions of the EQ-5D separately. The association between HRQoL and having experience with severe disease and/or having worked/working in health care could directly impact best practice of applied cost-utility analyses, for instance for interventions targeted at health care workers. General age-specific population norms may not apply to these groups to assess the impact of a specific disease on their quality of life evaluation. Furthermore, there is ongoing debate about the inclusion of caregivers’ HRQoL valuations (e.g [32–34]), and our findings show that if one chooses to include caregivers’ HRQoL impact (for instance HRQoL impact on parents, due to a disease in their child) general population norms may not apply to them.

Persons living in larger households (up to 5 persons) reported on average better HRQoL scores. This effect was not consistent between all imputed datasets, so future studies need to confirm this effect.

Unlike previous studies [5, 35], no association with gender was found in any of our analyses.

Children with only one parent have been shown to score lower on a psychosocial scale, but not on a physical scale [35], but no effect on EQ-5D or VAS score was found in our study.

Frequent contact with children and grandchildren may have a positive impact on happiness and HRQoL of elderly, but could also be experienced as a burden if it occurs too frequently, or could indicate reverse causality (where people who are sicker, receive more of these visits because of their illness). The fact that no overall effect of contacts with (grand)children was found on HRQoL in our study may be due to a mixture of all three reasons above, and is consistent with the findings of Muñoz-Pérez and Zapator-Torras [36].

Note that although we investigated 34 potential determinants of HRQoL, we may still have missed some important ones such as income (e.g. [37]), which we did not attempt to collect in our survey.

The population norms we produced for Belgium based on the EQ-5D index score are similar to the ones based on older surveys [1, 2], except that Szende et al. [1] estimated a higher average EQ-5D index score for older persons (e.g. for persons aged 65–74: 0.74–0.80 (our study) and 0.75–0.78 [2] compared to 0.85 [1]). Our population norms based on VAS are similar to the ones reported by Szende et al. [1] for young age groups but are higher for the older age groups (e.g. for persons aged 65–74: 81 [70–90] (our study, model including only age) and 79 [54–88] (our study, model including all significant determinants) compared to 71 [69–74] [1]). Our model for VAS including only age as a covariate failed to fit the data well for children and people aged 80 years or more. We were limited in the type of models to fit by age because of using beta regression models. Indeed, fitting non-linear models for outcomes with a beta error distribution is not a readily available option in standard statistical software. As a consequence, the relationship between HRQoL and age is not captured very well for all ages and should be used cautiously. Therefore, we also present population norms based on the models including all significant covariates. An alternative to the polynomials could be using splines to model HRQoL as a function of age (cfr.[38, 39]); however, exploratory analysis fitting polynomials, fractional polynomials and splines did not show a better fit of the spline models, which is why we decided to stick with the polynomial models (e.g. cfr. [40]). Another potential limitation is that when using our estimates for evaluating the cost-effectiveness of interventions prolonging life, this may result in an underestimation of HRQoL gained: i.e. poorer HRQoL at older age can be due to lower HRQoL preceding death, and when death is postponed through an intervention, also the period of lower HRQoL preceding death can be postponed. This can be dealt with by for instance modelling HRQoL as a function of time-to-death [41]. However, the empirical requirements to enable this in practice seem daunting.

We used (one-inflated) beta regression models, which capture some of the specific features of HRQoL variables. However, this implies assuming HRQoL to have a continuous distribution, whereas the EQ-5D-3L score has 243 possible states, and hence is by nature a categorical variable. Also, our analysis is based on a cross-sectional survey, hence not accounting for possible changes over time in HRQoL. There is much room for improvement in the way the widely used EQ-5D score is modelled, and indeed, this field of probability theory is very active (e.g. [39, 42–44]). Furthermore, we used the same dataset to identify predictors and the optimal function between HRQoL and age, as well as to re-categorize predictors, possibly resulting in over-fitting. A replication of our statistical analysis on an independent dataset is needed to investigate the generalizability of our findings.

In conclusion, we provide population norms for HRQoL (VAS and EQ-5D) by age in years. Additionally, we confirmed the importance of age, educational attainment, pet ownership and smoking behaviour in defining HRQoL and we are the first to show that HRQoL is potentially associated with witnessing severe disease and working or having worked in health care. Estimating HRQoL in children and developing statistical tools capturing the particular features of HRQoL measures are important areas for future research.

## Electronic supplementary material

Below is the link to the electronic supplementary material.

Supplementary material 1 (DOCX 204080 KB)
