# Supplementary material for: Quality-of-life: a many-splendored thing? Belgian population norms and 34 potential determinants explored by beta regression
Source: Qual Life Res. 2017 Mar 27;26(8):2011–23. doi: 10.1007/s11136-017-1556-y (PMC5509833; doi:10.1007/s11136-017-1556-y)
Supplement: Supplementary file 1 — Supplementary material 1 (DOCX 204080 KB) [file 11136_2017_1556_MOESM1_ESM.docx]

**Electronic Supplementary Material**

**Quality of Life: a Many-Splendored Thing? Belgian Population Norms and 34 Potential Determinants Explored by Beta Regression.**

**Journal:** Quality of Life Research

**Authors and affiliations**: Joke Bilcke^1^ (PhD), Niel Hens^1,2^ (PhD) and Philippe Beutels^1,3^ (PhD)

^1^Centre for Health Economics Research and Modeling Infectious Diseases (CHERMID), Vaccine and Infectious Disease Institute (VAXINFECTIO), University of Antwerp, Wilrijk, Belgium

^2^Institute for Biostatistics and statistical Bioinformatics (I-BioStat), Hasselt University, Diepenbeek, Belgium

^3^School of Public Health and Community Medicine, The University of New South Wales, Sydney, Australia

**Corresponding author**: joke.bilcke@uantwerp.be

**Content**

**Tables**

S1 Table. Pooled estimates for the final beta regression model for VAS score and one-inflated beta regression model for EQ-5D score as a function of age in years.

S2 Table. Estimated VAS and EQ-5D index scores as a function of age in years.

**Figures**

Fig. S1 VAS score as a function of 9 categorical determinants.

Fig. S2 EQ-5D index score (average score if not in perfect health) as a function of 9 categorical determinants.

Fig. S3 VAS score as a function of age in years only.

Fig. S4 EQ-5D index score as a function of age in years only.

Fig. S5 Diagnostic plots: QQ-plots.

Fig. S6 Diagnostic plots: randomized quantile residuals plotted against age.

**Results of all analyses without the data from children**

S1 Text Table 1: Pooled estimates for the final beta regression model for VAS score including 9 determinants, based on the data from adults and elderly only.

S1 Text Table 2: Pooled estimates for the final one-inflated beta regression model for EQ-5D index score including 12 determinants, based on the data from adults and elderly only.

S1 Text Table 3: Observed and fitted health-related quality-of-life scores and probabilities as a function of significant categorical variables, for adults and elderly only.

S1 Text Table 4: Pooled estimates for the final beta regression model for VAS score and one-inflated beta regression model for EQ-5D score as a function of age in years, based on the data from adults and elderly only.

S1 Text Table 5: Estimated VAS and EQ-5D index scores (+ 95% confidence intervals) as a function of age in years, for adults and elderly only.

**Survey data**

**Tables**

**S1 Table. Pooled estimates for the final beta regression model for VAS score and one-inflated beta regression model for EQ-5D score as a function of age in years.**

|  | pooled estimate | ll | ul | within-imputation variance | between-imputation variance | total variance | proportion variation  due to non-response |
| --- | --- | --- | --- | --- | --- | --- | --- |
| VAS score |  |  |  |  |  |  |  |
| Intercept | 2.24 | 2.17 | 2.30 | 0.002 | 0-0.0001 | 0.002 | 0.02% |
| age | -0.02 | -0.02 | -0.01 | 0-0.0001 | 0-0.0001 | 0-0.0001 | 0.07% |
| EQ-5D index score (nu model) |  |  |  |  |  |  |  |
| Intercept | 1.47 | 1.18 | 1.79 | 0.02 | 0.00064 | 0.03 | 3% |
| age | -0.0008 | -0.02 | 0.01 | 0-0.0001 | 0-0.0001 | 0-0.0001 | 2% |
| age^2^ | -0.0004 | -0.0006 | -0.0002 | 0-0.0001 | 0-0.0001 | 0-0.0001 | 1% |
| EQ-5D index score (mu model) |  |  |  |  |  |  |  |
| Intercept | 1.39 | 1.10 | 1.68 | 0.02 | 0.0009 | 0.02 | 5% |
| age | 0.02 | 0.009 | 0.03 | 0-0.0001 | 0-0.0001 | 0-0.0001 | 3% |
| age^2^ | -0.0003 | -0.0005 | -0.0002 | 0-0.0001 | 0-0.0001 | 0-0.0001 | 2% |

**S2 Table. Estimated VAS and EQ-5D index scores as a function of age in years.** Mean VAS and EQ-5D scores (on their original scale) and upper and lower limits of 95% confidence interval based on bootstrapping (including both within- and between-imputation variance) are presented, estimated based on age only or on all significant determinants.

|  | VAS score | | | | | | EQ-5D index score | | | | | |
| --- | --- | --- | --- | --- | --- | --- | --- | --- | --- | --- | --- | --- |
|  | estimated based on age | | | estimated based on all significant determinants | | | estimated based on age | | | estimated based on all significant determinants | | |
| age | mean | ll | ul | mean | ll | ul | mean | ll | ul | mean | ll | ul |
| 0 | 84 | 70 | 91 | 91 | 66 | 94 | 0.93 | 0.92 | 0.95 | 0.92 | 0.85 | 0.96 |
| 1 | 84 | 70 | 91 | 91 | 64 | 94 | 0.94 | 0.92 | 0.95 | 0.92 | 0.79 | 0.96 |
| 2 | 84 | 70 | 91 | 89 | 61 | 94 | 0.94 | 0.92 | 0.95 | 0.92 | 0.80 | 0.97 |
| 3 | 84 | 70 | 90 | 91 | 61 | 94 | 0.94 | 0.92 | 0.95 | 0.94 | 0.84 | 0.97 |
| 4 | 84 | 70 | 90 | 92 | 64 | 95 | 0.94 | 0.92 | 0.95 | 0.95 | 0.86 | 0.97 |
| 5 | 84 | 70 | 90 | 90 | 62 | 94 | 0.94 | 0.92 | 0.95 | 0.94 | 0.85 | 0.97 |
| 6 | 84 | 70 | 90 | 93 | 89 | 95 | 0.94 | 0.92 | 0.95 | 0.95 | 0.89 | 0.97 |
| 7 | 84 | 70 | 90 | 91 | 58 | 94 | 0.94 | 0.92 | 0.95 | 0.94 | 0.78 | 0.97 |
| 8 | 84 | 70 | 90 | 93 | 89 | 95 | 0.94 | 0.93 | 0.95 | 0.95 | 0.87 | 0.97 |
| 9 | 84 | 70 | 90 | 93 | 88 | 95 | 0.94 | 0.93 | 0.95 | 0.95 | 0.87 | 0.97 |
| 10 | 84 | 70 | 90 | 93 | 91 | 95 | 0.94 | 0.93 | 0.95 | 0.95 | 0.93 | 0.97 |
| 11 | 84 | 70 | 90 | 93 | 90 | 95 | 0.94 | 0.93 | 0.95 | 0.95 | 0.89 | 0.97 |
| 12 | 84 | 70 | 90 | 90 | 69 | 95 | 0.94 | 0.93 | 0.95 | 0.93 | 0.72 | 0.97 |
| 13 | 84 | 70 | 90 | 87 | 79 | 90 | 0.94 | 0.93 | 0.95 | 0.97 | 0.91 | 0.99 |
| 14 | 84 | 70 | 90 | 85 | 67 | 90 | 0.94 | 0.93 | 0.95 | 0.96 | 0.86 | 0.99 |
| 15 | 83 | 70 | 90 | 85 | 78 | 90 | 0.94 | 0.93 | 0.94 | 0.96 | 0.90 | 0.98 |
| 16 | 84 | 70 | 90 | 86 | 82 | 90 | 0.94 | 0.93 | 0.94 | 0.97 | 0.94 | 0.99 |
| 17 | 83 | 70 | 90 | 85 | 56 | 91 | 0.93 | 0.93 | 0.94 | 0.95 | 0.76 | 0.99 |
| 18 | 83 | 70 | 90 | 87 | 83 | 91 | 0.93 | 0.93 | 0.94 | 0.96 | 0.94 | 0.98 |
| 19 | 83 | 70 | 90 | 85 | 68 | 90 | 0.93 | 0.93 | 0.94 | 0.95 | 0.87 | 0.99 |
| 20 | 83 | 70 | 90 | 85 | 76 | 91 | 0.93 | 0.93 | 0.94 | 0.95 | 0.84 | 0.99 |
| 21 | 83 | 70 | 90 | 86 | 78 | 91 | 0.93 | 0.92 | 0.94 | 0.96 | 0.90 | 0.99 |
| 22 | 83 | 70 | 90 | 85 | 78 | 91 | 0.93 | 0.92 | 0.94 | 0.95 | 0.89 | 0.98 |
| 23 | 83 | 70 | 90 | 82 | 68 | 91 | 0.93 | 0.92 | 0.94 | 0.93 | 0.81 | 0.98 |
| 24 | 83 | 70 | 90 | 84 | 78 | 90 | 0.93 | 0.92 | 0.94 | 0.94 | 0.88 | 0.98 |
| 25 | 83 | 70 | 90 | 83 | 69 | 88 | 0.93 | 0.92 | 0.94 | 0.92 | 0.79 | 0.96 |
| 26 | 83 | 70 | 90 | 83 | 74 | 88 | 0.93 | 0.92 | 0.94 | 0.93 | 0.84 | 0.96 |
| 27 | 83 | 70 | 90 | 83 | 67 | 89 | 0.93 | 0.92 | 0.93 | 0.93 | 0.81 | 0.97 |
| 28 | 83 | 70 | 90 | 82 | 72 | 88 | 0.93 | 0.92 | 0.93 | 0.93 | 0.85 | 0.97 |
| 29 | 83 | 70 | 90 | 82 | 56 | 89 | 0.92 | 0.92 | 0.93 | 0.92 | 0.76 | 0.97 |
| 30 | 83 | 70 | 90 | 82 | 56 | 89 | 0.92 | 0.91 | 0.93 | 0.92 | 0.75 | 0.96 |
| 31 | 83 | 70 | 90 | 82 | 58 | 88 | 0.92 | 0.91 | 0.93 | 0.92 | 0.76 | 0.97 |
| 32 | 83 | 70 | 90 | 82 | 60 | 88 | 0.92 | 0.91 | 0.93 | 0.91 | 0.78 | 0.97 |
| 33 | 83 | 70 | 90 | 83 | 69 | 88 | 0.92 | 0.91 | 0.93 | 0.92 | 0.80 | 0.97 |
| 34 | 83 | 70 | 90 | 84 | 75 | 89 | 0.92 | 0.91 | 0.93 | 0.92 | 0.82 | 0.96 |
| 35 | 83 | 70 | 90 | 83 | 74 | 89 | 0.91 | 0.91 | 0.92 | 0.92 | 0.82 | 0.96 |
| 36 | 83 | 70 | 90 | 84 | 75 | 88 | 0.91 | 0.90 | 0.92 | 0.93 | 0.88 | 0.96 |
| 37 | 83 | 70 | 90 | 82 | 64 | 88 | 0.91 | 0.90 | 0.92 | 0.90 | 0.74 | 0.96 |
| 38 | 83 | 70 | 90 | 83 | 67 | 89 | 0.91 | 0.90 | 0.92 | 0.91 | 0.81 | 0.96 |
| 39 | 83 | 70 | 90 | 82 | 62 | 88 | 0.91 | 0.90 | 0.92 | 0.90 | 0.82 | 0.96 |
| 40 | 83 | 70 | 90 | 83 | 66 | 89 | 0.90 | 0.89 | 0.91 | 0.91 | 0.78 | 0.96 |
| 41 | 82 | 70 | 90 | 82 | 61 | 88 | 0.90 | 0.89 | 0.91 | 0.90 | 0.74 | 0.95 |
| 42 | 82 | 70 | 90 | 84 | 61 | 89 | 0.90 | 0.89 | 0.91 | 0.90 | 0.67 | 0.96 |
| 43 | 82 | 70 | 90 | 81 | 59 | 89 | 0.90 | 0.89 | 0.91 | 0.89 | 0.76 | 0.96 |
| 44 | 82 | 70 | 90 | 83 | 71 | 89 | 0.89 | 0.88 | 0.91 | 0.89 | 0.78 | 0.95 |
| 45 | 82 | 70 | 90 | 77 | 54 | 89 | 0.89 | 0.88 | 0.90 | 0.84 | 0.63 | 0.95 |
| 46 | 82 | 70 | 90 | 84 | 75 | 88 | 0.89 | 0.88 | 0.90 | 0.90 | 0.77 | 0.95 |
| 47 | 82 | 70 | 90 | 82 | 65 | 88 | 0.89 | 0.87 | 0.90 | 0.88 | 0.77 | 0.95 |
| 48 | 82 | 70 | 90 | 81 | 57 | 89 | 0.88 | 0.87 | 0.89 | 0.87 | 0.70 | 0.95 |
| 49 | 82 | 70 | 90 | 83 | 74 | 88 | 0.88 | 0.87 | 0.89 | 0.88 | 0.69 | 0.95 |
| 50 | 82 | 70 | 90 | 80 | 62 | 88 | 0.88 | 0.86 | 0.89 | 0.85 | 0.71 | 0.93 |
| 51 | 82 | 70 | 90 | 82 | 73 | 89 | 0.87 | 0.86 | 0.88 | 0.87 | 0.76 | 0.93 |
| 52 | 82 | 70 | 90 | 81 | 58 | 88 | 0.87 | 0.86 | 0.88 | 0.86 | 0.67 | 0.94 |
| 53 | 82 | 70 | 90 | 78 | 37 | 87 | 0.86 | 0.85 | 0.88 | 0.83 | 0.53 | 0.93 |
| 54 | 82 | 70 | 90 | 78 | 54 | 87 | 0.86 | 0.85 | 0.87 | 0.82 | 0.61 | 0.93 |
| 55 | 82 | 70 | 90 | 81 | 68 | 88 | 0.86 | 0.84 | 0.87 | 0.85 | 0.66 | 0.93 |
| 56 | 82 | 70 | 90 | 76 | 60 | 86 | 0.85 | 0.84 | 0.86 | 0.82 | 0.65 | 0.91 |
| 57 | 82 | 70 | 90 | 81 | 72 | 88 | 0.85 | 0.83 | 0.86 | 0.84 | 0.74 | 0.92 |
| 58 | 82 | 70 | 90 | 78 | 55 | 87 | 0.84 | 0.83 | 0.85 | 0.81 | 0.56 | 0.93 |
| 59 | 81 | 70 | 90 | 79 | 70 | 86 | 0.84 | 0.82 | 0.85 | 0.81 | 0.68 | 0.93 |
| 60 | 81 | 70 | 90 | 82 | 75 | 86 | 0.83 | 0.82 | 0.84 | 0.85 | 0.70 | 0.93 |
| 61 | 81 | 70 | 90 | 81 | 73 | 87 | 0.83 | 0.81 | 0.84 | 0.84 | 0.74 | 0.91 |
| 62 | 81 | 70 | 90 | 81 | 63 | 87 | 0.82 | 0.81 | 0.83 | 0.85 | 0.69 | 0.93 |
| 63 | 81 | 70 | 90 | 81 | 72 | 86 | 0.81 | 0.80 | 0.83 | 0.83 | 0.69 | 0.92 |
| 64 | 81 | 70 | 90 | 78 | 67 | 87 | 0.81 | 0.79 | 0.82 | 0.80 | 0.66 | 0.92 |
| 65 | 81 | 70 | 90 | 80 | 71 | 85 | 0.80 | 0.79 | 0.82 | 0.80 | 0.69 | 0.88 |
| 66 | 81 | 70 | 90 | 79 | 56 | 86 | 0.80 | 0.78 | 0.81 | 0.81 | 0.69 | 0.91 |
| 67 | 81 | 70 | 90 | 77 | 54 | 85 | 0.79 | 0.77 | 0.81 | 0.80 | 0.63 | 0.90 |
| 68 | 81 | 70 | 90 | 79 | 69 | 86 | 0.78 | 0.77 | 0.80 | 0.80 | 0.68 | 0.90 |
| 69 | 81 | 70 | 90 | 78 | 66 | 88 | 0.78 | 0.76 | 0.79 | 0.78 | 0.63 | 0.90 |
| 70 | 81 | 70 | 90 | 79 | 71 | 86 | 0.77 | 0.75 | 0.79 | 0.79 | 0.67 | 0.91 |
| 71 | 81 | 70 | 90 | 79 | 71 | 88 | 0.76 | 0.74 | 0.78 | 0.79 | 0.66 | 0.89 |
| 72 | 81 | 70 | 90 | 78 | 65 | 86 | 0.75 | 0.73 | 0.77 | 0.79 | 0.67 | 0.90 |
| 73 | 81 | 70 | 90 | 79 | 71 | 85 | 0.75 | 0.72 | 0.77 | 0.80 | 0.67 | 0.88 |
| 74 | 81 | 70 | 90 | 77 | 64 | 86 | 0.74 | 0.72 | 0.76 | 0.75 | 0.59 | 0.85 |
| 75 | 81 | 70 | 90 | 78 | 67 | 85 | 0.73 | 0.71 | 0.75 | 0.77 | 0.62 | 0.88 |
| 76 | 81 | 70 | 90 | 79 | 71 | 85 | 0.72 | 0.70 | 0.75 | 0.77 | 0.62 | 0.86 |
| 77 | 80 | 70 | 90 | 78 | 70 | 85 | 0.71 | 0.69 | 0.74 | 0.76 | 0.61 | 0.88 |
| 78 | 80 | 70 | 90 | 75 | 55 | 84 | 0.71 | 0.68 | 0.73 | 0.72 | 0.46 | 0.84 |
| 79 | 80 | 70 | 90 | 73 | 60 | 85 | 0.70 | 0.67 | 0.72 | 0.70 | 0.55 | 0.84 |
| 80 | 80 | 70 | 90 | 72 | 53 | 84 | 0.69 | 0.66 | 0.72 | 0.69 | 0.57 | 0.84 |
| 81 | 80 | 69 | 90 | 72 | 66 | 78 | 0.68 | 0.65 | 0.71 | 0.68 | 0.57 | 0.77 |
| 82 | 80 | 69 | 90 | 74 | 67 | 80 | 0.67 | 0.64 | 0.70 | 0.72 | 0.59 | 0.80 |
| 83 | 80 | 69 | 90 | 71 | 61 | 79 | 0.66 | 0.62 | 0.69 | 0.67 | 0.55 | 0.78 |
| 84 | 80 | 69 | 90 | 66 | 55 | 75 | 0.65 | 0.61 | 0.69 | 0.62 | 0.52 | 0.70 |
| 85 | 80 | 69 | 90 | 68 | 40 | 82 | 0.64 | 0.60 | 0.68 | 0.66 | 0.49 | 0.80 |
| 86 | 80 | 68 | 90 | 73 | 63 | 82 | 0.63 | 0.59 | 0.67 | 0.64 | 0.52 | 0.74 |
| 87 | 80 | 68 | 90 | 68 | 37 | 81 | 0.62 | 0.58 | 0.66 | 0.66 | 0.47 | 0.80 |
| 88 | 80 | 68 | 90 | 63 | 50 | 77 | 0.61 | 0.56 | 0.65 | 0.61 | 0.41 | 0.70 |
| 89 | 80 | 67 | 90 | 63 | 50 | 76 | 0.60 | 0.55 | 0.65 | 0.61 | 0.45 | 0.71 |
| 90 | 80 | 67 | 90 | 66 | 49 | 81 | 0.59 | 0.54 | 0.64 | 0.57 | 0.39 | 0.72 |
| 91 | 80 | 67 | 90 | 65 | 57 | 75 | 0.58 | 0.52 | 0.63 | 0.60 | 0.50 | 0.68 |
| 92 | 79 | 66 | 90 | 68 | 56 | 80 | 0.57 | 0.51 | 0.62 | 0.63 | 0.51 | 0.73 |
| 93 | 79 | 66 | 90 | 60 | 46 | 73 | 0.56 | 0.50 | 0.61 | 0.54 | 0.41 | 0.67 |
| 94 | 79 | 66 | 90 | 58 | 48 | 68 | 0.54 | 0.48 | 0.60 | 0.51 | 0.44 | 0.58 |
| 95 | 79 | 65 | 90 | 58 | 46 | 68 | 0.53 | 0.47 | 0.60 | 0.58 | 0.48 | 0.68 |

**Figures**

**Fig. S1** **VAS score as a function of 9 categorical determinants**. Boxplots of observed and predicted (blue) scores on their original scale. Boxplots of predicted VAS scores represent within- and between-imputation variance and are based on the final beta regression model containing 10 determinants (see Table 2).

**Fig. S2** **EQ-5D index score (average score if not in perfect health) as a function of 9 categorical determinants**. Boxplots of observed and predicted (blue) scores on their original scale. Boxplots of predicted EQ-5D scores represent within- and between-imputation variance and are based on the final one-inflated beta regression model for the EQ-5D index score containing 10 determinants (mu model, see Table 3).

**Fig. S3** **VAS score as a function of age in years only**. Boxplots of observed and predicted (blue) VAS scores on their original scale. Boxplots of predicted VAS scores represent within- and between-imputation variance and are based on the final beta regression model presented in S1 Table.

**Fig. S4** **EQ-5D index score as a function of age in years only.** Boxplots of observed and predicted (blue) EQ-5D index scores on their original scale. Boxplots of predicted EQ-5D index scores represent within- and between-imputation variance and are based on the final one-inflated beta regression model presented in S1 Table.

**Fig. S5 Diagnostic plots: QQ-plots.** QQ-plots of the normalized randomized quantile residuals for the four final models based on one of the five imputed datasets (plots for the five imputed datasets are very similar). Topleft: QQ-plot for one-inflated beta regression model of EQ-5D index score as a function of 10 (mu model) and 7 (nu model) determinants; topright: QQ-plot for beta regression model of VAS score as a function of 10 determinants; bottomleft: QQ-plot for one-inflated beta regression model of EQ-5D index score as a function of age only; bottomright: QQ-plot for beta regression model of VAS score as a function of age only.

**Fig. S6 Diagnostic plots: randomized quantile residuals plotted against age.** Plots for the four final models based on one of the five imputed datasets (plots for the five imputed datasets are very similar). Topleft: Plot for one-inflated beta regression model of EQ-5D index score as a function of 10 (mu model) and 7 (nu model) determinants; topright: Plot for beta regression model of VAS score as a function of 10 determinants; bottomleft: Plot for one-inflated beta regression model of EQ-5D index score as a function of age only; bottomright: Plot for beta regression model of VAS score as a function of age only.

**Results of all analyses without the data from children**

**S1 Text - Table 1**. **Pooled estimates for the final beta regression model for VAS score including 9 determinants, based on the data from adults and elderly only.**

|  | pooled estimate | ll | ul | within-imputation variance | between-imputation variance | total variance | proportion variation  due to non-response |
| --- | --- | --- | --- | --- | --- | --- | --- |
| Intercept | 1.46 | 0.98 | 1.92 | 0.06 | 0.005 | 0.07 | 9% |
| age | 0.02 | 0.003 | 0.04 | 0-0.0001 | 0-0.0001 | 0-0.0001 | 10% |
| age squared | -0.0003 | -0.0005 | -0.0001-0 | 0-0.0001 | 0-0.0001 | 0-0.0001 | 14% |
| experience severe disease with oneself | -0.43 | -0.57 | -0.30 | 0.005 | 0.0003 | 0.005 | 7% |
| experience severe disease with family member | -0.24 | -0.36 | -0.11 | 0.003 | 0.0007 | 0.004 | 21% |
| pets | -0.13 | -0.24 | -0.02 | 0.003 | 0.0003 | 0.003 | 9% |
| sick | -0.88 | -1.12 | -0.63 | 0.02 | 0.0007 | 0.02 | 5% |
| disabled | -0.80 | -1.04 | -0.57 | 0.01 | 0.0009 | 0.01 | 7% |
| work(ed) in health sector | 0.16 | 0.03 | 0.29 | 0.004 | 0.0004 | 0.004 | 11% |
| education level: none or primary school | -0.20 | -0.37 | -0.01 | 0.009 | 0.001 | 0.01 | 16% |
| education level: higher (not-) university | 0.02 | -0.09 | 0.14 | 0.003 | 0.0002 | 0.004 | 7% |
| education level: student | 0.36 | 0.05 | 0.67 | 0.02 | 0.001 | 0.03 | 6% |
| elderly | 0.07 | -0.15 | 0.30 | 0.01 | 0.0008 | 0.01 | 7% |
| living in elderly home or with family | -0.35 | -0.71 | 0.06 | 0.03 | 0.009 | 0.04 | 28% |

**S1 Text - Table 2.** **Pooled estimates for the final one-inflated beta regression model for the EQ-5D index score including 12 determinants, based on the data from adults and elderly only.**

|  | | | pooled estimate | ll | ul | within-imputation variance | between-imputation variance | total variance | proportion variation due to non-response |
| --- | --- | --- | --- | --- | --- | --- | --- | --- | --- |
|  |  | Nu model for the probability to be in perfect health (i.e. EQ-5D index score=1) | | | | | | | |
| Intercept | | | 0.69 | -0.35 | 1.77 | 0.29 | 0.003 | 0.29 | 1% |
| age | | | 0.05 | 0.01 | 0.09 | 0.0004 | 0-0.0001 | 0.0004 | 0.8% |
| age squared | | | -0.0008 | -0.001 | -0.0004 | 0-0.0001 | 0-0.0001 | 0-0.0001 | 0.7% |
| sick | | | -1.96 | -3.00 | -1.01 | 0.25 | 0.005 | 0.26 | 3% |
| experience severe disease with oneself | | | -1.02 | -1.35 | -0.71 | 0.02 | 0.002 | 0.03 | 9% |
| experience severe disease with family member | | | -0.57 | -0.85 | -0.31 | 0.02 | 0.0005 | 0.02 | 3% |
| pets | | | -0.29 | -0.56 | -0.03 | 0.02 | 0.0001 | 0.02 | 0.8% |
| education level: none or primary school | | | -0.91 | -1.46 | -0.39 | 0.07 | 0.001 | 0.07 | 2% |
| education level: higher (not-) university | | | 0.44 | 0.17 | 0.72 | 0.02 | 0.0002 | 0.02 | 1% |
| education level: student | | | 1.24 | 0.54 | 2.04 | 0.15 | 0.0006 | 0.15 | 0.5% |
| work(ed) in health sector | | | -0.36 | -0.67 | -0.06 | 0.02 | 0.0002 | 0.02 | 1% |
|  |  | Mu model for the average EQ-5D score if not in perfect health | | | | | | | |
| Intercept | | | 1.08 | 0.71 | 1.49 | 0.04 | 0.005 | 0.04 | 14% |
| experience severe disease with oneself | | | -0.19 | -0.37 | -0.03 | 0.007 | 0.0006 | 0.007 | 10% |
| experience severe disease with family member | | | 0.15 | -0.04 | 0.34 | 0.008 | 0.002 | 0.01 | 27% |
| household size | | | 0.21 | 0.03 | 0.44 | 0.009 | 0.0009 | 0.01 | 10% |
| household size squared | | | -0.02 | -0.06 | 0.007 | 0.0002 | 0-0.0001 | 0.0002 | 17% |
| adult | | | 0.08 | -0.09 | 0.24 | 0.007 | 0.0002 | 0.008 | 3% |
| disabled | | | -1.02 | -1.37 | -0.68 | 0.03 | <0.0001 | 0.03 | 0.3% |
| smoking | | | -0.30 | -0.52 | -0.09 | 0.01 | 0.0001 | 0.01 | 1% |
| no experience severe disease because of caring for someone | | | 0.20 | -0.03 | 0.42 | 0.01 | 0.002 | 0.01 | 15% |
| work(ed) in health sector | | | 0.17 | 0.009 | 0.34 | 0.007 | 0.001 | 0.008 | 18% |
| sick | | | -0.41 | -0.89 | 0.09 | 0.06 | 0.002 | 0.06 | 3% |
| living in elderly home or with family | | | -1.59 | -1.98 | -1.23 | 0.04 | 0-0.0001 | 0.04 | 0.2% |

**S1 Text - Table 3.** **Observed and fitted health-related quality-of-life scores and probabilities as a function of significant categorical variables, for adults and elderly only.** Mean observed scores en fitted means with 95% confidence intervals reflecting both within- and between-imputation variance. Fitted values for VAS and EQ-5D are given on their original scale.

|  |  | VAS | | EQ-5D probability perfect health | | EQ-5D if not in perfect health | |
| --- | --- | --- | --- | --- | --- | --- | --- |
|  |  | data | model | data | model | data | model |
| experience severe disease with oneself | no | 83 | 83 [68-89] | 0.68 | 0.67 [0.17-0.92] | 0.69 | 0.69 [0.58-0.73] |
|  | yes | 71 | 71 [49-82] | 0.32 | 0.31 [0.02-0.67] | 0.58 | 0.62 [0.34-0.71] |
| experience severe disease with family member | no | 83 | 84 [62-90] | 0.73 | 0.70 [0.10-0.93] | 0.63 | 0.67 [0.37-0.73] |
|  | yes | 80 | 79 [56-87] | 0.56 | 0.53 [0.05-0.86] | 0.67 | 0.68 [0.43-0.73] |
| experience severe disease because of caring for someone | no |  |  |  |  | 0.67 | 0.68 [0.45-0.73] |
|  | yes |  |  |  |  | 0.63 | 0.64 [0.34-0.72] |
| going to (regular) school | no |  |  |  |  |  |  |
|  | yes |  |  |  |  |  |  |
| smoking | no |  |  |  |  | 0.70 | 0.70 [0.60-0.73] |
|  | yes |  |  |  |  | 0.61 | 0.65 [0.46-0.71] |
| drinking alcohol | no or rarely |  |  |  |  |  |  |
|  | yes |  |  |  |  |  |  |
| disabled | no | 81 | 81 [61-89] |  |  | 0.66 | 0.68 [0.41-0.73] |
|  | yes | 61 | 60 [41-73] |  |  | 0.47 | 0.51 [0.38-0.62] |
| work(ed) in health sector | no | 80 | 80 [56-88] | 0.61 | 0.61 [0.06-0.92] | 0.64 | 0.67 [0.40-0.73] |
|  | yes | 82 | 83 [61-90] | 0.57 | 0.56 [0.07-0.86] | 0.69 | 0.69 [0.48-0.74] |
| highest education level | none or primary | 70 | 69 [45-85] | 0.23 | 0.22 [0.01-0.63] |  |  |
|  | secondary | 80 | 80 [58-88] | 0.54 | 0.53 [0.09-0.81] |  |  |
|  | higher education | 83 | 82 [68-89] | 0.69 | 0.68 [0.25-0.88] |  |  |
|  | student | 85 | 86 [78-91] | 0.86 | 0.86 [0.64-0.95] |  |  |
| pets | no | 80 | 80 [54-89] | 0.58 | 0.57 [0.04-0.91] |  |  |
|  | yes | 81 | 81 [61-89] | 0.61 | 0.61 [0.12-0.91] |  |  |
| sick | no | 81 | 81 [59-89] | 0.61 | 0.60 [0.07-0.91] | 0.65 | 0.68 [0.41-0.73] |
|  | yes | 65 | 64 [36-78] | 0.26 | 0.26 [0.01-0.66] | 0.59 | 0.63 [0.35-0.72] |
| living in elderly home or with family | no | 78 | 78 [62-86] |  |  | 0.67 | 0.67 [0.61-0.72] |
|  | yes | 61 | 60 [43-75] |  |  | 0.36 | 0.38 [0.24-0.48] |

**S1 Text - Table 4. Pooled estimates for the final beta regression model for VAS score and one-inflated beta regression model for EQ-5D score, based on the data from adults and elderly only.**

|  | pooled estimate | ll | ul | within-imputation variance | between-imputation variance | total variance | proportion variation  due to non-response |
| --- | --- | --- | --- | --- | --- | --- | --- |
| VAS score |  |  |  |  |  |  |  |
| Intercept | 1.99 | 1.83 | 2.15 | 0.007 | 0.0002 | 0.007 | 4% |
| age | -0.01 | -0.01 | -0.009 | 0-0.0001 | 0-0.0001 | 0-0.0001 | 3% |
| EQ-5D index score (nu model) |  |  |  |  |  |  |  |
| Intercept | 1.65 | 1.01 | 2.32 | 0.12 | 0.0002 | 0.12 | 0.2% |
| age | -0.008 | -0.03 | 0.02 | 0.0002 | 0-0.0001 | 0.0002 | 0.2% |
| age^2^ | -0.0003 | -0.0006 | -0.0001-0 | 0-0.0001 | 0-0.0001 | 0-0.0001 | 0.2% |
| EQ-5D index score (mu model) |  |  |  |  |  |  |  |
| Intercept | 0.75 | 0.20 | 1.25 | 0.07 | 0.0003 | 0.07 | 0.5% |
| age | 0.04 | 0.02 | 0.07 | 0.0001 | 0-0.0001 | 0.0001 | 1% |
| age^2^ | -0.0005 | -0.0007 | -0.0003 | 0-0.0001 | 0-0.0001 | 0-0.0001 | 0.9% |

**S1 Text - Table 5. Estimated VAS and EQ-5D index scores as a function of age in years, for adults and elderly only.** Mean VAS and EQ-5D scores (on their original scale) and upper and lower limits of 95% confidence interval based on bootstrapping (including both within- and between-imputation variance) are presented, estimated based on age only or on all significant determinants.

|  | VAS score | | | | | | EQ-5D index score | | | | | |
| --- | --- | --- | --- | --- | --- | --- | --- | --- | --- | --- | --- | --- |
|  | estimated based on age | | | estimated based on all significant determinants | | | estimated based on age | | | estimated based on all significant determinants | | |
| age | mean | ll | ul | mean | ll | ul | mean | ll | ul | mean | ll | ul |
| 13 | 86 | 85 | 88 | 87 | 79 | 90 | 0.93 | 0.91 | 0.95 | 0.97 | 0.90 | 0.99 |
| 14 | 86 | 85 | 88 | 85 | 68 | 90 | 0.93 | 0.91 | 0.95 | 0.95 | 0.80 | 0.98 |
| 15 | 86 | 85 | 87 | 85 | 77 | 90 | 0.93 | 0.91 | 0.95 | 0.96 | 0.89 | 0.98 |
| 16 | 86 | 84 | 87 | 86 | 82 | 90 | 0.93 | 0.91 | 0.95 | 0.96 | 0.94 | 0.98 |
| 17 | 86 | 84 | 87 | 85 | 56 | 91 | 0.93 | 0.91 | 0.95 | 0.94 | 0.65 | 0.99 |
| 18 | 86 | 84 | 87 | 87 | 83 | 91 | 0.93 | 0.91 | 0.95 | 0.96 | 0.93 | 0.98 |
| 19 | 85 | 84 | 87 | 85 | 68 | 91 | 0.93 | 0.91 | 0.94 | 0.94 | 0.81 | 0.98 |
| 20 | 85 | 84 | 87 | 86 | 76 | 91 | 0.93 | 0.91 | 0.94 | 0.94 | 0.81 | 0.99 |
| 21 | 85 | 84 | 86 | 86 | 79 | 91 | 0.93 | 0.91 | 0.94 | 0.95 | 0.88 | 0.99 |
| 22 | 85 | 84 | 86 | 86 | 78 | 91 | 0.93 | 0.91 | 0.94 | 0.95 | 0.87 | 0.98 |
| 23 | 85 | 84 | 86 | 83 | 67 | 91 | 0.93 | 0.91 | 0.94 | 0.92 | 0.73 | 0.98 |
| 24 | 85 | 83 | 86 | 85 | 79 | 90 | 0.93 | 0.91 | 0.94 | 0.93 | 0.86 | 0.98 |
| 25 | 85 | 83 | 86 | 84 | 70 | 89 | 0.93 | 0.91 | 0.94 | 0.91 | 0.71 | 0.96 |
| 26 | 84 | 83 | 86 | 83 | 74 | 88 | 0.93 | 0.91 | 0.94 | 0.92 | 0.83 | 0.96 |
| 27 | 84 | 83 | 85 | 83 | 68 | 89 | 0.92 | 0.91 | 0.93 | 0.92 | 0.78 | 0.97 |
| 28 | 84 | 83 | 85 | 83 | 73 | 88 | 0.92 | 0.91 | 0.93 | 0.92 | 0.83 | 0.97 |
| 29 | 84 | 83 | 85 | 83 | 57 | 89 | 0.92 | 0.91 | 0.93 | 0.92 | 0.71 | 0.97 |
| 30 | 84 | 83 | 85 | 82 | 56 | 89 | 0.92 | 0.91 | 0.93 | 0.91 | 0.71 | 0.96 |
| 31 | 84 | 82 | 85 | 83 | 58 | 88 | 0.92 | 0.91 | 0.93 | 0.91 | 0.72 | 0.97 |
| 32 | 83 | 82 | 84 | 82 | 60 | 88 | 0.92 | 0.91 | 0.93 | 0.90 | 0.73 | 0.97 |
| 33 | 83 | 82 | 84 | 83 | 68 | 88 | 0.92 | 0.91 | 0.93 | 0.91 | 0.76 | 0.97 |
| 34 | 83 | 82 | 84 | 84 | 75 | 89 | 0.92 | 0.91 | 0.92 | 0.92 | 0.81 | 0.96 |
| 35 | 83 | 82 | 84 | 83 | 74 | 89 | 0.91 | 0.90 | 0.92 | 0.91 | 0.81 | 0.96 |
| 36 | 83 | 82 | 84 | 84 | 74 | 89 | 0.91 | 0.90 | 0.92 | 0.93 | 0.88 | 0.96 |
| 37 | 83 | 82 | 84 | 82 | 65 | 88 | 0.91 | 0.90 | 0.92 | 0.89 | 0.67 | 0.97 |
| 38 | 82 | 81 | 83 | 83 | 67 | 89 | 0.91 | 0.90 | 0.92 | 0.91 | 0.78 | 0.96 |
| 39 | 82 | 81 | 83 | 82 | 62 | 88 | 0.91 | 0.90 | 0.92 | 0.90 | 0.80 | 0.96 |
| 40 | 82 | 81 | 83 | 83 | 66 | 89 | 0.90 | 0.89 | 0.91 | 0.91 | 0.73 | 0.97 |
| 41 | 82 | 81 | 83 | 82 | 62 | 88 | 0.90 | 0.89 | 0.91 | 0.89 | 0.70 | 0.95 |
| 42 | 82 | 81 | 83 | 84 | 62 | 89 | 0.90 | 0.89 | 0.91 | 0.91 | 0.65 | 0.96 |
| 43 | 82 | 81 | 82 | 81 | 58 | 89 | 0.90 | 0.89 | 0.91 | 0.90 | 0.75 | 0.96 |
| 44 | 81 | 80 | 82 | 83 | 71 | 89 | 0.89 | 0.88 | 0.90 | 0.90 | 0.77 | 0.96 |
| 45 | 81 | 80 | 82 | 76 | 54 | 89 | 0.89 | 0.88 | 0.90 | 0.84 | 0.63 | 0.96 |
| 46 | 81 | 80 | 82 | 83 | 74 | 88 | 0.89 | 0.88 | 0.90 | 0.90 | 0.78 | 0.95 |
| 47 | 81 | 80 | 82 | 82 | 66 | 88 | 0.89 | 0.87 | 0.90 | 0.88 | 0.75 | 0.96 |
| 48 | 81 | 80 | 81 | 81 | 57 | 89 | 0.88 | 0.87 | 0.89 | 0.88 | 0.71 | 0.95 |
| 49 | 80 | 80 | 81 | 83 | 75 | 88 | 0.88 | 0.87 | 0.89 | 0.89 | 0.71 | 0.96 |
| 50 | 80 | 79 | 81 | 80 | 62 | 87 | 0.88 | 0.86 | 0.89 | 0.86 | 0.72 | 0.94 |
| 51 | 80 | 79 | 81 | 83 | 74 | 89 | 0.87 | 0.86 | 0.88 | 0.88 | 0.78 | 0.94 |
| 52 | 80 | 79 | 81 | 81 | 57 | 88 | 0.87 | 0.86 | 0.88 | 0.87 | 0.69 | 0.95 |
| 53 | 80 | 79 | 81 | 78 | 38 | 87 | 0.86 | 0.85 | 0.88 | 0.84 | 0.51 | 0.94 |
| 54 | 80 | 79 | 80 | 78 | 55 | 87 | 0.86 | 0.85 | 0.87 | 0.83 | 0.64 | 0.95 |
| 55 | 79 | 78 | 80 | 81 | 67 | 88 | 0.86 | 0.84 | 0.87 | 0.86 | 0.71 | 0.95 |
| 56 | 79 | 78 | 80 | 77 | 60 | 86 | 0.85 | 0.84 | 0.87 | 0.84 | 0.67 | 0.93 |
| 57 | 79 | 78 | 80 | 81 | 72 | 87 | 0.85 | 0.83 | 0.86 | 0.86 | 0.77 | 0.93 |
| 58 | 79 | 78 | 80 | 78 | 56 | 87 | 0.84 | 0.83 | 0.86 | 0.83 | 0.60 | 0.95 |
| 59 | 79 | 78 | 80 | 79 | 71 | 87 | 0.84 | 0.82 | 0.85 | 0.84 | 0.74 | 0.94 |
| 60 | 78 | 77 | 79 | 82 | 75 | 86 | 0.83 | 0.82 | 0.85 | 0.87 | 0.74 | 0.94 |
| 61 | 78 | 77 | 79 | 81 | 73 | 86 | 0.83 | 0.81 | 0.84 | 0.84 | 0.74 | 0.91 |
| 62 | 78 | 77 | 79 | 80 | 62 | 87 | 0.82 | 0.81 | 0.84 | 0.85 | 0.64 | 0.94 |
| 63 | 78 | 77 | 79 | 81 | 72 | 86 | 0.82 | 0.80 | 0.83 | 0.84 | 0.71 | 0.92 |
| 64 | 78 | 76 | 79 | 78 | 67 | 87 | 0.81 | 0.80 | 0.83 | 0.80 | 0.69 | 0.93 |
| 65 | 77 | 76 | 79 | 80 | 71 | 85 | 0.81 | 0.79 | 0.82 | 0.81 | 0.72 | 0.90 |
| 66 | 77 | 76 | 78 | 79 | 56 | 86 | 0.80 | 0.78 | 0.82 | 0.81 | 0.64 | 0.91 |
| 67 | 77 | 76 | 78 | 77 | 53 | 85 | 0.79 | 0.78 | 0.81 | 0.79 | 0.59 | 0.90 |
| 68 | 77 | 75 | 78 | 79 | 70 | 86 | 0.79 | 0.77 | 0.80 | 0.81 | 0.68 | 0.91 |
| 69 | 76 | 75 | 78 | 78 | 65 | 88 | 0.78 | 0.76 | 0.80 | 0.80 | 0.69 | 0.91 |
| 70 | 76 | 75 | 78 | 79 | 71 | 86 | 0.77 | 0.76 | 0.79 | 0.80 | 0.71 | 0.92 |
| 71 | 76 | 75 | 78 | 79 | 71 | 88 | 0.77 | 0.75 | 0.79 | 0.81 | 0.71 | 0.91 |
| 72 | 76 | 74 | 77 | 78 | 65 | 86 | 0.76 | 0.74 | 0.78 | 0.80 | 0.69 | 0.91 |
| 73 | 76 | 74 | 77 | 79 | 71 | 86 | 0.75 | 0.73 | 0.77 | 0.80 | 0.65 | 0.89 |
| 74 | 75 | 74 | 77 | 77 | 65 | 87 | 0.74 | 0.72 | 0.76 | 0.78 | 0.67 | 0.87 |
| 75 | 75 | 74 | 77 | 78 | 67 | 85 | 0.74 | 0.72 | 0.76 | 0.79 | 0.68 | 0.89 |
| 76 | 75 | 73 | 77 | 79 | 72 | 85 | 0.73 | 0.71 | 0.75 | 0.80 | 0.69 | 0.89 |
| 77 | 75 | 73 | 77 | 78 | 70 | 85 | 0.72 | 0.70 | 0.74 | 0.79 | 0.68 | 0.89 |
| 78 | 75 | 73 | 76 | 75 | 55 | 84 | 0.71 | 0.69 | 0.73 | 0.75 | 0.54 | 0.86 |
| 79 | 74 | 72 | 76 | 73 | 59 | 86 | 0.70 | 0.67 | 0.73 | 0.73 | 0.41 | 0.86 |
| 80 | 74 | 72 | 76 | 72 | 54 | 84 | 0.69 | 0.66 | 0.72 | 0.71 | 0.43 | 0.88 |
| 81 | 74 | 72 | 76 | 72 | 67 | 79 | 0.68 | 0.65 | 0.71 | 0.70 | 0.49 | 0.81 |
| 82 | 74 | 72 | 76 | 74 | 67 | 80 | 0.67 | 0.64 | 0.70 | 0.75 | 0.69 | 0.81 |
| 83 | 73 | 71 | 76 | 71 | 61 | 79 | 0.66 | 0.63 | 0.69 | 0.72 | 0.66 | 0.79 |
| 84 | 73 | 71 | 75 | 66 | 55 | 76 | 0.65 | 0.62 | 0.69 | 0.66 | 0.42 | 0.74 |
| 85 | 73 | 71 | 75 | 69 | 42 | 83 | 0.64 | 0.60 | 0.68 | 0.66 | 0.40 | 0.84 |
| 86 | 73 | 70 | 75 | 73 | 62 | 83 | 0.63 | 0.59 | 0.67 | 0.72 | 0.64 | 0.81 |
| 87 | 72 | 70 | 75 | 69 | 38 | 82 | 0.62 | 0.58 | 0.66 | 0.65 | 0.39 | 0.83 |
| 88 | 72 | 70 | 75 | 63 | 49 | 78 | 0.61 | 0.56 | 0.65 | 0.51 | 0.36 | 0.73 |
| 89 | 72 | 69 | 74 | 64 | 51 | 76 | 0.60 | 0.55 | 0.64 | 0.51 | 0.31 | 0.71 |
| 90 | 72 | 69 | 74 | 66 | 48 | 81 | 0.58 | 0.53 | 0.63 | 0.52 | 0.34 | 0.79 |
| 91 | 72 | 69 | 74 | 64 | 56 | 76 | 0.57 | 0.51 | 0.62 | 0.47 | 0.36 | 0.58 |
| 92 | 71 | 69 | 74 | 68 | 56 | 81 | 0.56 | 0.50 | 0.62 | 0.58 | 0.36 | 0.76 |
| 93 | 71 | 68 | 74 | 60 | 46 | 75 | 0.55 | 0.48 | 0.61 | 0.44 | 0.31 | 0.59 |
| 94 | 71 | 68 | 74 | 59 | 49 | 68 | 0.53 | 0.47 | 0.60 | 0.40 | 0.30 | 0.51 |
| 95 | 71 | 68 | 73 | 57 | 45 | 68 | 0.52 | 0.45 | 0.59 | 0.52 | 0.36 | 0.68 |

**Survey data**

These are available upon request from the authors.
